# Physiological and pathological implications of 5-hydroxymethylcytosine in diseases

**DOI:** 10.18632/oncotarget.9281

**Published:** 2016-05-10

**Authors:** Jing Liang, Fan Yang, Liang Zhao, Chongwei Bi, Benzhi Cai

**Affiliations:** ^1^ Department of Pharmacology, Harbin Medical University (The State-Province Key Laboratories of Biomedicine-Pharmaceutics of China), Harbin, China; ^2^ Institute of Clinical Pharmacy and Medicine, Academics of Medical Sciences of Heilongjiang Province, Harbin, China

**Keywords:** 5hmC, TET, methylation, embryogenesis, heart

## Abstract

Gene expression is the prerequisite of proteins. Diverse stimuli result in alteration of gene expression profile by base substitution for quite a long time. However, during the past decades, accumulating studies proved that bases modification is involved in this process. CpG islands (CGIs) are DNA fragments enriched in CpG repeats which mostly locate in promoters. They are frequently modified, methylated in most conditions, thereby suggesting a role of methylation in profiling gene expression. DNA methylation occurs in many conditions, such as cancer, embryogenesis, nervous system diseases etc. Recently, 5-hydroxymethylcytosine (5hmC), the product of 5-methylcytosine (5mC) demethylation, is emerging as a novel demethylation marker in many disorders. Consistently, conversion of 5mC to 5hmC has been proved in many studies. Here, we reviewed recent studies concerning demethylation *via* 5hmC conversion in several conditions and progress of therapeutics-associated with it in clinic. We aimed to unveil its physiological and pathological significance in diseases and to provide insight into its clinical application potential.

## INTRODUCTION

**Figure 1:**
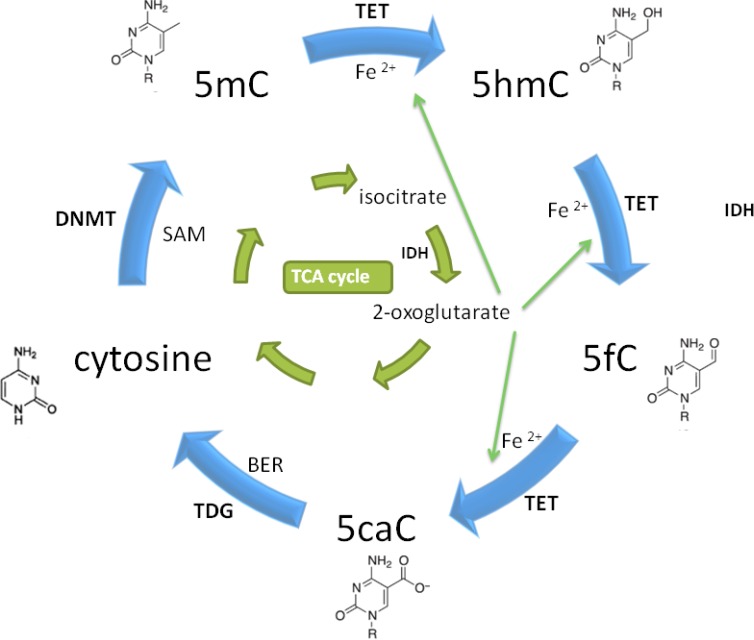
Introduction and elimination of 5hmC TCA: tricarboxylic acid; BER: base excision repair; SAM: S-Adenosylmethionine.

Epigenetics defines a group of nucleotides modification, such DNA methylation, microRNA interference etc, as well as histone change, such as histone acetylation, methylation, phosphorylation etc, which could switch gene expression on or off. Chemical modification rather than base substitution occurs within DNA bases during epigenetic modification. DNA methylation is one of the most widely studied epigenetic modifications, and the critical role of DNA methylation in various conditions has been largely illustrated [1–3]. This process is precisely regulated by several DNA methyltransferases (DNMTs) including DNMT1, DNMT3A and DNMT3B. These enzymes are committed to transferring a methyl group from S-adenosyl methionine (SAM) to the 5^th^ carbon of the cytosine which results in methylation. DNMT1 is maintenance methyltransferase which functions to retaining methylation status after early embryonic phase [4]. DNMT3A/B is de novo methyltransferase which regulates methylation status during embryogenesis [5]. 5mC is one of the cytosine derivatives, and other derivatives include 5hmC, 5-formylcytosine (5fC) and 5-carboxycytosine (5caC). 5mC, the dominant methylated product, constitutes ~1% of all mammalian DNA bases [6]. 5hmC, the product of 5mC hydroxylation, is involved in methyl group elimination and supposed to be an essential demethylation intermediate [7, 8]. 5mC hydroxylation is catalyzed by Fe (II)-and 2-oxoglutarate (2-OG)-dependent dioxygenase ten-eleven-translocation (TETs) [8–10] (Figure 1). It has been proved that DNA methylation frequently happens in repetitive DNA fragments enriched in CGIs of inter-and intra-genes, such as tumor suppressor genes (TSG). It suggests the potential correlation between methylation and diseases, and provides some new targets for diseases treatment [11–14]. Thus, demethylation via 5hmC conversion probably abrogates methylation, thus ameliorating diseases. Remarkably, some studies have identified that re-patterning of 5hmC profile did exist under diverse pathologic and/or physiologic conditions while 5hmC recovery could reverse them [15–19]. In this review, we mainly focus on demethylation via 5hmC conversion in cancer, embryogenesis, nervous system disorder as well as cardiovascular system, and attempt to uncover its role in poorly understood areas. Meanwhile, perspective of its clinical application is also discussed to present its fantastic future.

## DEMETHYLATION VIA 5HMC CONVERSION IN CANCER

Cancer which is capable of invading other tissues refers to disease concerning abnormal cell growth and proliferation. Although a large body of studies have highlighted that lots of stimuli, genetic and environmental mainly, may contribute to carcinogenesis limited studies mention epigenetics [20–23]. Recently, considerable studies have suggested involvement of epigenetics in cancer [24–27]. Meanwhile, it was reported that diverse epigenetic modifications were implicated in carcinogenesis in which methylation plays a leading role. In most cancer studies, methylation dysregulation followed by gene silencing or activation often occurred in pivotal genes, such as TSGs and oncogenes [28–32]. Hence, demethylation of these sites perhaps eradicates cancer. 5hmC, the primary product of DNA demethylation, was firstly discovered in mammals by Penn NW et al in 1972 [33]. Then it was found implicated in carcinogenesis [34–37]. Global loss of 5hmC was observed in large numbers of cancer in which TETs dysregulation was detected conincidentally [33, 38].

### 1. Demethylation *via* 5hmC conversion in hematopoietic malignancies

#### i. Leukemia

DNA or chromosomal abnormalities is able to induce leukemia. Oncogenes, TSGs and other genes frequently mutate in leukemia [39–41]. Meanwhile, aberrant rearrangement of chromosomes is also suggested a role in leukemia [42, 43]. TET2 involvement in leukemia has been largely demonstrated. F Viguié et al manifested that gene encoding TET2 was rearranged and deleted in acute myeloid leukemia (AML). It may indicate impairment of 5mC hydroxylation and subsequent 5hmC reduction in several studies [18, 44, 45]. Because of the key role of TETs in demethylation, regulators of TETs are capable of regulating demethylation. Isocitrate dehydrogenase (IDH) is able to catalyze 2-OG production in citric cycle. IDH mutation disturbs catalytic activity of TETs by producing 2-HG instead of 2-OG, thus inducing hypermethylation in leukemic patients [45]. A clinical study revealed that leukemic patients with both TET and IDH mutation showed lower 5hmC compared with those without TET or IDH mutation. Meanwhile, they manifested patients with high 5hmC showed lower overall survival which indicated some other pathways might involve leukemia [46]. Recently, a study suggested that IDH2/R140Q mutation decreased demethylation *via* 5hmC conversion and increased expression of several differentiation-related genes accompanied with activation of Meis1-related hypoxia pathway in transgenic leukemic mice [47]. It indicates critical role of IDH2 in development and maintenance of AML stem cells and implication of environmental factors in leukemia. However, Sadudee Chotirat et al. concluded a subtle role of IDH mutation in preleukemic disorder which implies rare involvement of IDH in leukemia at the initial stage [48]. In addition, mutation of WT1, which binds to TET2 and cooperatively recruits to target site, was detected in interruption of DNA demethylation associated with TET2 in AML. It suggested the direct interaction between WT1 and demethylation mediated by TET2 and provided a novel therapeutic target [49–51]. Interestingly, another study showed that hypoxia was involved in decrease of DNMT and TET2/TET3 along with increase of demethylation and WT1 expression. Again, it incorporated environmental factors into conditions associated with demethylation [47, 52]. Additionally, TETs were suggested to bind to and be regulated by CRL4^VprBP^ which monoubiquitylated its partners and offered them access to chromatin [53].

In conclusion, accumulating regulators of TETs are being discovered and proved as potential targets in leukemia treatment. Besides, environmental implication in leukemia is also suggested. It proposes the notion that both epigenetic modification and environment are involved in leukemogenesis.

**Figure 2:**
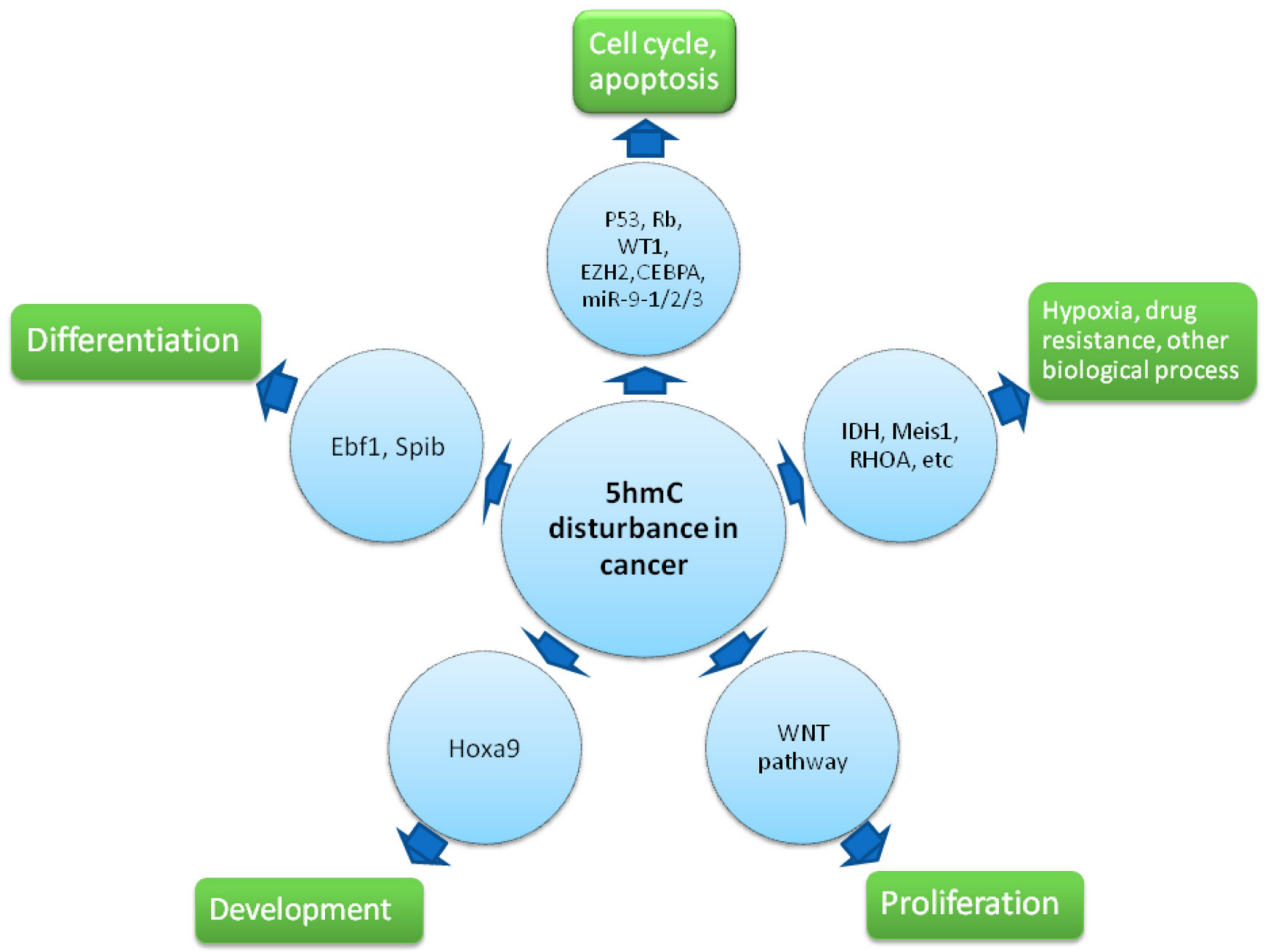
Effect of 5hmC disturbance upon cancer 5hmC regulates cell cycle progress, apoptosis, differentiation, proliferation and development *via* their critical regulator like TSGs and oncogenes, Ebf1, Spib, Hoxa9, etc. Furthermore, 5hmC is implicated activation of Meis1-associated hypoxia pathway, RHOA-associated biological process as well as other pathways.

#### ii. Lymphoma

Lymphoma is lymphocyte-derived lymphatic tumor. It constitutes around 55% of all hematologic malignancies. Although diverse lymphocytes are involved in lymphoma, two main subtypes, T-cell and B-cell lymphomas are exclusively focused. In contrast to TETs disruption in other tumors, TETs disruption is closely associated with lymphoma [54, 55].

Cyril Quivoron et al showed that TET2 mutation was involved in human B-cell and T-cell lymphoma [56]. Another study of genomic profiling in diffuse large B-cell lymphoma found TET2 mutation primarily correlated with hypermethylation of hematopoietic differentiation-and development-associated genes. Among these genes, only 11% were decreased and several tumor suppressors were included [57]. A large body of studies evidenced the key role of TET2 in most tumors and TET1 mutation was also suggested as tumor suppressor in B-cell lymphoma [54]. Lately, a study demonstrated that TET1 deletion was able to induce B-cell lymphoma. Gene sequencing showed that the mutation dominant in TET1-deleted mice was observed in non-Hodgkin B cell lymphoma (B-NHL). It indicated the crucial role of TET1 as tumor suppressor in B-NHL as well as a novel potential target for its treatment [58]. To date, these conclusions drawn from B-cell lymphoma studies provide more targets and improve prognosis of diseases.

T-cell lymphoma is associated with TETs as well [59–61]. In a study of human T-cell lymphoma, simultaneous mutation of TET2 and DNMT3A was detected and DNMT3A mutation precedes TET2 mutation. It indicated the role of DNMT3A in T-cell lymphoma at early stage [61]. Consistently, a case study showed that TET2 was mutated in most samples (~76% in cohort) along with mutation of other genes, such as IDH2 and DNMT3A [62]. Besides, mutation of RHOA was also found following TET2 mutation. It suggested that TET2 might regulate physiology-associated processes through demethylation [63].

#### iii. Myeloma

Myeloma constitutes ~14% of total hematologic malignancies. It is involved in multiple organs and tissues, such as head, bone, kidney, blood etc, and thus being designated as multiple myelomas (MMs). Methylation influences myeloma by suppressing TSGs, activating oncogenes or regulating other proliferation-and differentiation-associated genes [64–66]. Although methylation is widely involved in MMs, relationship between MMs and demethylation *via* 5hmC conversion is poorly studied. Tumor suppressors, miR-9-1 and miR-9-3, were showed to be re-activated by 5-aza-2′-deoxycytidine in MMs. This suggests a new participator of MMs pathogenesis [67]. DNMT3A was downregulated resulting from hypermethylation. It suggested methylation abnormality happened in MMs [68]. Thus, DNMT3A is nominated as a prognostic marker of MMs. Moreover, Xabier Agirre et al observed that different hypermethylated sites were showed in enhancers other than promoters. It implied genes downregulation was correlated with enhancers hypermethylation in MMs. Therefore, it enables us to focus on other elements of genes besides promoters [69]. Though there are few studies associated with MMs, studies of DNMTs-mediated methylation dysregulation forward the research on TETs in MMs.

### Demethylation *via* 5hmC conversion in alimentary system malignancies

Alimentary system consists of mouth, esophagus, stomach, intestine and their accessory organs including pancreas, liver and cholecyst. Rich blood vessels, body fluid transportation as well as environmental factors predispose alimentary system to malignancy. Hence, it is very important to unravel detailed mechanisms. Increasing importance of methylation manifested in cancers makes demethylation a novel way of anti-cancer.

#### i. Esophageal cancer

Though esophagus is positioned as the initial portion of alimentary system, few studies mentioned interrelationship between demethylation *via* 5hmC conversion and esophageal cancer. Recently, Asuka Murata et al. showed that 5hmC was decreased in esophageal squamous cell carcinoma (ESCC), and this reduction was due to TET2 suppression. Meanwhile, a direct correlation between 5hmC and overall survival (OS) was also manifested in this study [70].

#### ii. Gastric cancer

Lots of studies highlighted that 5hmC loss in gastric cancer (GC) was associated with TET1 downregulation [71, 72]. Accordingly, other studies identified that 5hmC was downregulated through decreasing TET1, not TET2 or TET3, and indicated low 5hmC as a poor diagnostic marker in GC [36, 73]. As tumor suppressor, TET1 suppression in GC was attributed to promoter hypermethylation of TET1. It further verified the disturbance of DNA methylation in cancer [74]. Consistently, Fu et al. showed that TET1 functioned as tumor suppressor *via* TSGs activation and oncogenes inhibition [75]. Herein, 5hmC dysregulation due to disturbance of various TETs indicates that any TET is probably involved in demethylation of GC.

#### iii. Colorectal cancer

Demethylation *via* 5hmC conversion in colorectal cancer (CRC) is largely proposed and studied. Considerable decrease of 5hmC in CRC was detected, and thus suggesting demethylation disturbance is involved in CRC [76]. Keun Hur et al revealed that hypomethylation was capable of activating several oncogenes which were silenced by methylation in CRC [77]. Although TETs mutation in CRC is rarely studied, alteration of their methylation is frequently addressed. Norihisa Ichimura et al. demonstrated TET1 methylation could contribute to CRC, thus aiding in CRC diagnosis [78]. Another study confirmed TET1 was involved in cancer cells proliferation via WNT signal pathway. Meanwhile, it suggested TET1 as tumor suppressor in colon cancer [79]. Besides, Santiago Uribe-Lewis et al. demonstrated that promoters enriched in 5hmC in normal colon as well as targets of TET2 in CRC were resistant to hypermethylation while this process was not mediated by TET2. Thus, it indicated 5hmC could be a biomarker for proliferation of cancer cells in CRC [80]. Moreover, TET1 was increased by ROS resulting from 5-FU followed by induction of cancer drug resistance in CRC. It indicates involvement of ROS pathway in demethylation and drug resistance [81, 82].

### 3. Demethylation *via* 5hmC conversion in nervous system tumors

Tumors in nervous system include glioblastoma, glioma, astrocytoma etc. As demethylation implication in other cancers, it is also associated with nervous system tumors. A study in normal brain and brain tumor showed that 5hmC was decreased to 1.1-1.9% in brain tumor compared with 61.5% in normal brain. It indicated 5hmC could be a marker of brain cancer [83]. It was consistent with studies presented by Jin SG and Brent A Orr [84, 85]. Meanwhile, the inverse relationship between 5hmC level and proliferation of brain tumor cells was concluded. It indicated the potential of 5hmC as a diagnosis marker of brain tumor. Mutant IDH1, which is profoundly related to methylome reconstruction of glioma, could produce 2-OG, and thus inhibiting TETs [86]. However, another study demonstrated that 5hmC loss was independent of IDH1 mutation but associated with nuclear exclusion of TETs [87, 88]. Thereby, it pushed us back to track. Consistently, Hans A Kretzschmar et al observed 5hmC increased in IDH1 mutation-independent way in glioblastoma [89]. It also showed the inverse proportion of 5hmC and proliferation markers, Ki67 and H3S10p. This indicated involvement of demethylation in brain tumor cells proliferation and potential of 5hmC as tumor marker. Notwithstanding, Kraus TF et al demonstrated that 5hmC disturbance in brain tumor was not associated with TETs mutation while it might be related to disruption of gene expression or inhibition of related proteins [90].

In addition to these cancers, other cancers, like melanoma, hepatocellular carcinoma, breast cancer etc, were also highlighted to be associated with demethylation disturbance [91–94]. One or more TETs are likely involved in cancers followed by products change of target genes which are related to proliferation, differentiation, tumorigenesis, drug resistance etc. Interference with these pathways provides us more possibility to eradicate cancers. Meanwhile, more targets are in the pipeline. They inspire researchers to design more specific and potent therapies for cancers.

## DEMETHYLATION VIA 5HMC CONVERSION IN EMBRYOGENESIS

Embryogenesis is a quite sophisticated process composed of cell proliferation and differentiation at early embryonic stage. After fertilization, zygote cleaves on the 2^nd^ day which will not stop until achieving a blastomere comprising 16 or 32 cells termed as morula. Subsequently, blastulation, which indicates several mitotic divisions making cells count to 128, occurs followed by blastula implantation, gastrulation and organogenesis in order. This is the so-called histological process of embryogenesis. However, how each cell is committed to the cell with specific functions, such as neuron, gland cell, and movement cell, is a totally different story. DNA imprinting is epigenetic modification of DNA existing in all somatic cells. It confers specific expression profile to individual cell followed by specific functions. It has been verified that DNA methylation is the major player in DNA imprinting [95, 96]. The genome-wide demethylation and the following methylation recovery occur at pre-implantation stage in primordial germ cells (PGCs). Preparation of this process for cell totipotency retrieval has been proved, thus identifying the role of demethylation in normal embryogenesis and suggesting the role of eccentric demethylation in abnormal embryogenesis [97, 98]. A large number of studies have showed that DNA methylation in embryogenesis was due to DNA methyltransferase, and demethylation was due to TETs [5, 99–104].

### 1. Demethylation *via* 5hmC conversion in pre-implantation reprogramming

During pre-implantation stage, cleaving zygote is demethylated after 6-8 hours of fertilization in paternal genome while several cell divisions in maternal genome. Thenceforth, cells undergo de novo methylation for gene expression profiling [95, 97]. Thus, pre-implantation reprogramming *via* demethylation and methylation is especially essential for embryogenesis.

Several pathways are implicated in demethylation of early embryogenesis and PGCs [105, 106]. 5mC conversion to 5hmC is considered as one genome-wide demethylation pathway in pre-implantation reprogramming, and several candidate demethylases are verified [96, 107]. Recently, several studies showed that TETs further oxidize 5hmC, 5fmC and 5caC stepwise whose end-product can be converted to 5C by thymine DNA glycosylase (TDG) or replication-dependent dilution at early embryonic phase [8, 108, 109]. Shinsuke Ito et al revealed that TET1 played an important role in demethylation of mouse embryogenesis *via* 5hmC conversion. They observed that TET1 KO could induce differentiation preference. It indicated the role of TET1 in embryonic cells specification and maintenance [17]. Another study confirmed the role of TET1 in active DNA demethylation *via* 5hmC conversion in early porcine embryo. It also demonstrated that 5mC, not 5hmC, was involved in initial cell specification in blastocysts [104]. However, an *in vivo* study showed that TET1 KO did not influence multipotency and embryogenesis [110]. Thus, different models may result in different results. Raul Mostoslavsky et al illustrated that histone deacetylase sirtuin 6 (SIRT6) KO was capable of increasing 5hmC in TET-dependent way. This drew out an upstream regulator of TETs-mediated demethylation. Furthermore, it implies that other epigenetic modifications, such as histone acetylation, are implicated in genome-wide demethylation through mediating TETs [111]. Besides, Jinsuk Kang et al revealed that much loss of 5hmC and much gain of 5mC due to TET1/3 KO in embryo of eight-cell stage occurred along with altered gene expression which could produce holoprosencephaly phenotype [112]. Meelad M Dawlaty et al found that triple-knockout of TET1/2/3 was able to diminish 5hmC. This impaired ESC specification in both embryonic stem cells (ESCs) and embryoid bodies (EB) by promoter hypermethylation [113]. However, another study exhibited different methylation style of DNA imprinting region in TETs-deleted models. It suggested that DNA imprinting regions of various genes were differentially modulated by TET. Furthermore, irregular methylation of DNA imprinting regions after TETs deletion observed in ESC and EB suggested limited application of EB in epigenetic study of embryogenesis [114]. Additionally, a study concluded that PRDM4 was capable of accelerating active demethylation which is partially through TET1 and TET2 recruitment to target loci in ESCs. It implied existence of other participators in TETs-mediated demethylation [115]. Taken together, TETs-mediated demethylation is crucial for embryogenesis. The involvement of this demethylation pathway in developmental abnormalities just started. More targets and the detailed mechanisms are required to be fully demonstrated.

### 2. Demethylation *via* 5hmC conversion in PGC reprogramming

PGC is the second extragonadal source of gamete cells occurring at 8^th^ day after gestation [116, 117]. PGC, which is capable of being induced to pluripotent stem cells (PSCs), derives from epiblast before inner cell mass (ICM) generation [118, 119]. Similar with pre-implantation, genome-wide DNA demethylation as well as methylation recovery occurs in PGC to obtain germ cell phenotype [120–122]. Genome demethylation is completed between E10.5 and E12.5 after gestation, and then the roles of multiple demethylases in PGC development are demonstrated [123, 124].

Recently, a study demonstrated that elevated TET1/2 decreased methylation in PGC, and sites escaping from demethylation, which were proved to be repeated loci, such as intracisternal A-particle (IAP), were also showed [102]. Thus, similar mechanism mediated by TETs may prevail in PGC reprogramming. Another study suggested that 5mC and 5hmC were low during E8.5 and E9.5 before 5hmC increased. The fact 5hmC enriched at chromatin area in TET1-dependent way implied TETs might be implicated in other modifications [125]. John J Vincent et al illustrated that TET1/2-mediated demethylation was involved in late phase of demethylation rather than initial phase. It suggested TETs played different roles in different phases of PGC reprogramming [126]. Another study concluded that DNA demethylation was correlated with ssDNA break and base excision repair pathways. It exhibited the potential function of DNA demethylation in PGC development [127]. Meanwhile, a study in AID deficiency implied other partners might play some roles in PGC methylation [128]. Calvopina Joseph Hargan demonstrated that demethylation pathway in PGC which was replication-dependent was similar with ESCs [129]. Therefore, TETs-mediated demethylation in PGC reprogramming plays the crucial role as in ESCs. They are differentially implicated in various phases of PGC reprogramming and target diverse signal pathways in addition to demethylation. To date, our insight into the role of demethylation *via* 5hmC conversion in PGC reprogramming is limited, and further studies are required.

## DEMETHYLATION VIA 5HMC CONVERSION IN NERVOUS SYSTEM

Methylation is involved in nervous system while demethylation is less addressed [130–138]. However, milestone built by Skirmantas Kriaucionis, Shinsuke Ito and Mamta Tahiliani changes this situation [8, 10, 139]. From then on, accumulating studies showed that demethylation *via* 5hmC conversion was correlated with development and diseases in nervous system. Though 5hmC is abundant in brain for obscure reason, it drives researchers to explore its roles in nervous system.

### 1. Demethylation *via* 5hmC conversion in nervous system development

5hmC, which constitutes 0.6% of all nucleotides in Purkinje neurons and 0.2% in granule cells, is involved in nervous system development [140]. It was showed that 8% CpG of all autosomal chromosomes contained 5hmC, and it mainly enriched in enhancers and exons [141]. Differential distribution of 5hmC in brain implied 5hmC was associated with neurodevelopment and disorders [142–144]. Keith E Szulwach et al demonstrated that dynamic shift of 5hmC occurred at different stages of neural cells. However, some sites were 5hmC-conservative. Besides, 5hmC was in inverse proportion with methyl-CpG-binding protein 2 which was Rett syndrome-associated protein [15]. It suggested 5hmC played roles in nervous system. Meanwhile, 5hmC was implicated in nervous system development in a positive correlation way. 5hmC enriched in exons and UTR while diminished in introns and intergenic regions. It was associated with autism [145]. Therefore, 5hmC does participate in nervous system development as well as nervous system diseases. Recently, Run-Rui Zhang et al demonstrated that TET1 KO could downregulate proliferation of neural progenitor cells followed by impairment of hippocampal neurogenesis in brain of adult mice which showed poor ability of learning and memory. They also detected increase of methylation and decrease of gene expression. These genes were correlated with proliferation of neural progenitor cells [146]. Particularly, a study detecting 5hmC among stem cells, neuronal progenitor cells and mature olfactory sensory neurons (mOSNs) found that 5hmC increased with development and was obviously abundant in mOSNs. Additionally, TET3 overexpression resulted in hypomethylation, increased gene expression as well as physiologic abnormality [147]. However, TET3 was showed to play a role in regulation of Dnmt3a and Dkk1 expression by demethylation, in neural stem cells [148]. Interestingly, Svetlana Dzitoyeva et al examined 5hmC in mitochondria of mouse frontal cortex and cerebellum at different ages. They found that aging was exclusively associated with 5hmC decrease while not with 5mC in frontal cortex. Moreover, mtDNMT1 was depleted while TET2/TET3 was unaffected in frontal cortex. Nonetheless, TET1/TET3 was elevated while mtDNMT1 was unaffected during aging in cerebellum [149]. Strikingly, an environmental study showed that vitamin C could induce neural stem cells to differentiate into another type of neurons related to Parkinson's disease. It was associated with increased 5hmC. Gene KO study suggested that it was mediated by TET1 [150]. This study, however, not only exhibited importance of demethylation in nervous system disorders, but emphasized effect of environmental factors on epigenetic modifications.

### 2. Demethylation *via* 5hmC conversion in other nervous system disorders

Besides nervous system development, demethylation via 5hmC conversion is emerging as a key epigenetic modification in other nervous disorders. 5hmC reduction was found in Huntington's disease (HD) which was mediated by several signal pathways associated with neural development and differentiation [151]. In Alzheimer's disease (AD), both 5mC and 5hmC were elevated along with differential distribution in various neural cells and increasing AD markers. It indicated targeting 5hmC could be applied in AD treatment [152]. Moreover, in Autism spectrum disorder (ASD), A Zhubi et al observed 5hmC enriched in promoter of methyl CpG binding protein-2 (MeCP2)-binding accompanied with TET1 enrichment and binding. They were able to facilitate MeCP2 binding and gene expression [153]. Furthermore, in prenatal stress, Erbo Dong et al. revealed that 5hmC enriched in promoter brain-derived neurotrophic factor (Bdnf) gene. This gene correlated with social behaviors. Thus, it implied a potential role of 5hmC in reducing Bdnf expression [154].

Overall, considerable studies showed the role of demethylation *via* 5hmC conversion in nervous system. Most of them clarified the involvement of TETs in this process while some did not. Therefore, more studies are required for its role in nervous system.

## DEMETHYLATION VIA 5HMC CONVERSION IN OTHER CONDITIONS

The particular fate of cardiomyocytes similar with neurons requires studies concentrating on demethylation in heart. Though 5hmC is very low in human heart, high 5hmC enrichment in repetitive elements and intergenic regions in cardiac hypertrophy compared with high 5hmC enrichment in gene bodies of normal heart suggests that 5hmC-mediated demethylation shifts under pathologic condition. It links heart dysfunction to demethylation [76, 155]. Another study revealed that a large scale of 5hmC enriched in Notch pathway-related genes in skeletal muscle, heart, and cerebellum. This pathway was critical for stem cells [156]. Recently, a study in uterus showed that caffeine exposure could alter methylation patterning by interrupting both DNMTs and TETs. This alteration increased wall thickness and left ventricular mass [157]. Consistently, cardiac hypertrophy induced by cAMP was through similar mechanism [158]. Renjing Liu et al showed that TET2 was capable of regulating plasticity of smooth muscle cells (SMCs) and differentiation of fibroblasts towards SMCs while TET2-dependent 5hmC upregulation could mitigate intimal hyperplasia [159]. It showed a potential role of demethylation *via* 5hmC conversion mediated by TETs in differentiation of cardiovascular cells. Otherwise, arsenic, a drug closely correlated with heart dysfunction, was showed to be associated with demethylation in rat heart. It suggested a novel mechanism of arsenic in heart diseases [160–164]. These studies extend our insight into the roles of demethylation in cardiovascular system and suggest a bright future of demethylation in heart diseases treatment.

**Figure 3:**
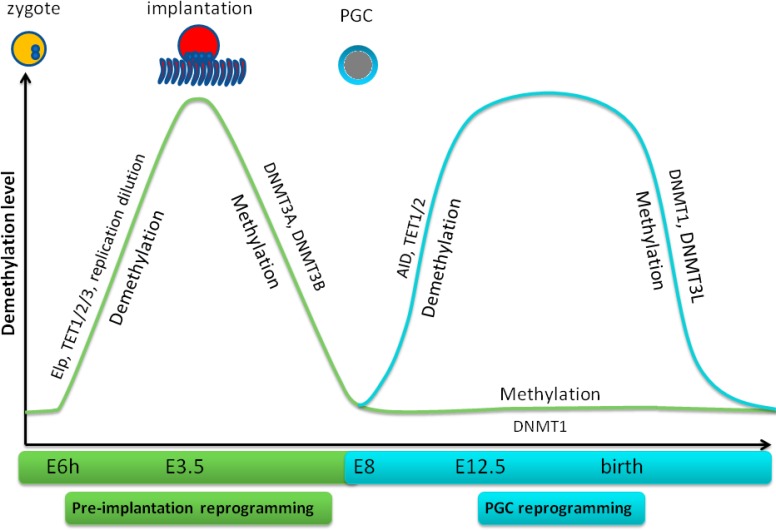
Demethylation and remethylation in mouse early embryogenesis and PGC reprogramming In pre-implantation reprogramming, demethylation initiates at 6h-8h after fertilization and is completed on E6 when implantation happens. Thereafter, remethylation initiates and completes around E9. Demethylation of PGC initiates on E10 and attains in 2 days while remethylation occurs at birth.

## PHAMACOTHERAPEUTICS OF DEMETHYLATION IN DISEASES

The important role of demethylation in diseases indicates the promising perspective of demethylation therapy. In fact, several demethylation agents have been studied and applied as a strategy for diseases treatment, such as 5-azacitidine, decitabine, zebularine, clofarabine, fazarabine, MG98 etc [165]. As newly discovered demethylation enzymes, candidates intervening TETs are in the pipeline. However, stories happened to former demethylation agents may promise us its encouraging perspective.

### 1. Demethylation agents targeting DNMTs

Different from TETs, DNMTs are important maintenance methyltransferase in mammalian cells. A lot of studies have showed that DNMTs imbalance occurs widely in various conditions and is closely linked to their abnormal methylation [166, 167]. DNMTs inhibition induces DNA demethylation. Agents targeting DNMTs have presented remarkable role clinically [168, 169, 170]. Meanwhile, DNMTs themself can be a clinical predictor of disease stages [167].

**Figure 4:**
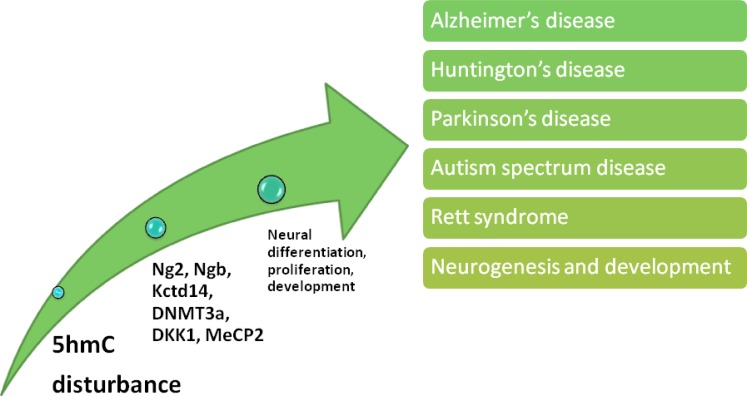
Effects of 5hmC disturbance upon nervous system 5hmC is involved in various nervous system disorders *via* transcription dysregulation of a large pool of factors, and these factors play essential role in neural cellular differentiation, proliferation and development.

#### i. Nucleoside analogues

Generally, the majority of DNMTs inhibitors are nucleoside analogues. These inhibitors can be incorporated into DNA or RNA followed by covalent bond formation with DNMTs and consequent inhibition [165]. Then, methylation profile will be altered in organisms.

5-azacitidine (5-AZA), one of cytidine analogues, has been approved by FDA as a drug mainly for MDS treatment in 2004. The approval summary reported that 5-AZA was able to improve prognosis of MDS patients by eliminating the transfusion dependence and normalization of blood cells as well as bone marrow morphology [171]. Notably, a recent clinic study demonstrated that 5-AZA showed effect in AML treatment [172]. This study evidenced that 5-AZA improved OS in contrast to conventional regimens. Furthermore, 5-AZA was more effective for patients with cytogenetics and MDS alterations. Besides, substantial studies have suggested the role of 5-AZA in various cancers [173, 174, 175]. Although demethylation activity of 5-AZA has been reported for quite a long time, its mechanism was considered to be mediated mainly through inhibiting RNA/DNA synthesis initially while its implication in DNMTs regulation was demonstrated later [176]. Decitabine, also called 5-aza-2′-deoxycytidine, is another analogue of cytidine which can be incorporated into DNA, and subsequently inhibiting DNMTs. Decitabine was approved as drug for MDS treatment by targeting DNMTs in 2006 [177]. A MDS patients study showed that patients receiving decitabine attained higher response rate, improved hematologic environment as well as longer interruption to deteriorative stages [178]. Similar with 5-AZA, decitabine shows effect in multiple diseases, such as AML, lymphoma, lung cancer etc [179, 180, 181]. Different from 5-AZA, decitabine is deoxynucleoside analogue which determines its exclusive role in DNA methylation. Thanks to 5-AZA and decitabine, zebularine, a new member of cytidine analogues, was synthesized aiming to provide a more effective and safer drug for cancer treatment. In addition to DNMT, zebularine also deactivates cytidine deaminase which is crucial for maintenance of pharmacologic activity of cytidine analogues, and thus improving its effect [182, 183]. Interestingly, Cheng JC et al. found that not only did zebularine confer cells growth inhibition and silenced cancer-antigen genes re-expression to cancer cells, but also zebularine downregulated DNMT1 preferentially [184]. Also, zebularine functions in multiple conditions including immune diseases, cell differentiation etc [185, 186].

Clofarabine, an adenosine analogue inspired from fludarabine and cladribine, is a FDA-approved drug for paediatric ALL in 2004 [187]. At first, mechanism of clofarabine effect is considered to be mediated mainly by three pathways: DNA polymerase inhibition, ribonucleotide reductase inhibition and apoptosis induction [188]. However, several recent studies manifested that clofarabine could demethylate cells at low concentration which subsequently induces re-expression of silenced genes in cancers [189, 190]. These findings indicate demethylation may be the 4^th^ pathway of clofarabine treatment in cancers which may be independent of DNMTs.

#### ii. Non-nucleoside analogues agent

MG 98, a 20bp antisense oligodeoxynucleotide, specifically binds to 3′-UTR of DNMT1, and subsequently interfering DNMT1 expression. Many studies of MG98 have been carried out in various scopes while different results are obtained in different conditions. Rebecca B. Klisovic et al. reported that MG98 showed no effect in AML treatment [191]. Nonetheless, Robert J. Amato et al suggested that combination of MG98 and interferon-α-2β was capable of alleviating advanced renal cell carcinoma [192]. These differences may result from cancer-selectivity and combination with other agents of MG98. Thus, it is still fair fantastic to investigate antisense oligonucleotide focusing on its role in varied conditions and its combination with other agents.

RG108 is a newly synthesized inhibitor which functions by directly binding to active site of DNMT1. Bodo Brueckner et al. evidenced that RG108 could demethylate cancer cells significantly followed by TSGs re-expression. Notably, RG108 treatment did not manifest any toxicity [193]. Another study also showed that RG108 was capable of inhibiting proliferation of cancer cells. In addition, it demonstrated that its combination with histone acetylation inhibitors could strengthen potency of RG108 [194]. Interestingly, RG108 could meliorate stem cells therapy by improving anti-senescence of stem cells [195]. As a new DNMT inhibitor, RG108 deserves considerable exploration due to its low toxicity and other advantages.

In sum, nucleoside analogues are predominant demethylation agents targeting DNMT. Many of them have showed remarkable perspective and been applied as clinic regimens. Nevertheless, side effects emerge as another challenge for us. For example, decitabine interrupts RNA, DNA and protein synthesis both in sick and healthy cells, and thus being toxic. Therefore, biochemical synthesis is required to develop more effective and safer agents. That is why agents, such as MG98 and RG108, are synthesized.

### 2. Demethylation agents targeting TET

As TETs are newly discovered methylation-associated enzymes, agents targeting them are on the way. However, several studies have given us insight into its bright future.

A study showed that vitamin C was able to induce TET-dependent demethylation in ES cells by activating TETs followed by genes re-expression. It suggested that agents targeting TET could alter stem cells destiny, and thus probably being applied in clinic [196]. In human cancer skin cells, vitamin C treatment conferred anti-apoptotic function to cells by activating TET, demethylating DNA, and thus re-patterning gene expression profile [197]. Additionally, vitamin C-mediated TET regulation was involved in nervous system development [150].

Although there is no specific agent targeting TETs due to their new membership in family of demethylation agents, what happens to DNMTs as well as great progress of TET studies inspires us to explore deeply and continuously.

## SUMMARY

Here, we summarized studies mainly pertaining to demethylation of 5hmC conversion mediated by TETs in several conditions as well as progress of TETs-associated therapeutics. We conclude that demethylation does play pivotal roles in cancer, embryogenesis, nervous system, CVS and other conditions despite poorly available studies in some categories, and these roles in these conditions are associated with proliferation, differentiation, DNA break repair, drug resistance and other process at transcription level. With these discoveries, 5hmC, similar with 5mC, is increasingly applied as a prognostic biomarker in disease advance and treatment. 5hmC reversing is capable of rectifying the disturbance resulting from methylation dysregulation in some diseases. To date, its effects in most cancers, embryogenesis and nervous system have been well established, but more studies about its role in other conditions like CVS are required. Moreover, researches of TETs-dependent demethylation agents are just at the beginning, more investigations are intensely required. Though there may be a long way, this way is fair fantastic and interesting.

## References

[1] Tuominen R, Jewell R, van den Oord JJ, Wolter P, Stierner U, Lindholm C, Hertzman Johansson C, Linden D, Johansson H, Frostvik Stolt M, Walker C, Snowden H, Newton-Bishop J, Hansson J, Egyhazi Brage S (2015). MGMT promoter methylation is associated with temozolomide response and prolonged progression-free survival in disseminated cutaneous melanoma. International journal of cancer.

[2] Arribas AJ, Rinaldi A, Mensah AA, Kwee I, Cascione L, Robles EF, Martinez-Climent JA, Oscier D, Arcaini L, Baldini L, Marasca R, Thieblemont C, Briere J, Forconi F, Zamo A, Bonifacio M (2015). DNA methylation profiling identifies two splenic marginal zone lymphoma subgroups with different clinical and genetic features. Blood.

[3] Castelo-Branco P, Choufani S, Mack S, Gallagher D, Zhang C, Lipman T, Zhukova N, Walker EJ, Martin D, Merino D, Wasserman JD, Elizabeth C, Alon N, Zhang L, Hovestadt V, Kool M (2013). Methylation of the TERT promoter and risk stratification of childhood brain tumours: an integrative genomic and molecular study. The Lancet Oncology.

[4] Goyal R, Reinhardt R, Jeltsch A (2006). Accuracy of DNA methylation pattern preservation by the Dnmt1 methyltransferase. Nucleic acids research.

[5] Okano M, Bell DW, Haber DA, Li E (1999). DNA methyltransferases Dnmt3a and Dnmt3b are essential for de novo methylation and mammalian development. Cell.

[6] Ehrlich M, Gama-Sosa MA, Huang LH, Midgett RM, Kuo KC, McCune RA, Gehrke C (1982). Amount and distribution of 5-methylcytosine in human DNA from different types of tissues of cells. Nucleic Acids Res.

[7] Guo JU, Su Y, Zhong C, Ming G-l, Song H (2011). Hydroxylation of 5-methylcytosine by TET1 promotes active DNA demethylation in the adult brain. Cell.

[8] Tahiliani M, Koh KP, Shen Y, Pastor WA, Bandukwala H, Brudno Y, Agarwal S, Iyer LM, Liu DR, Aravind L, Rao A (2009). Conversion of 5-methylcytosine to 5-hydroxymethylcytosine in mammalian DNA by MLL partner TET1. Science (New York, N Y).

[9] Tan L, Shi YG (2012). Tet family proteins and 5-hydroxymethylcytosine in development and disease. Development (Cambridge, England).

[10] Ito S, Shen L, Dai Q, Wu SC, Collins LB, Swenberg JA, He C, Zhang Y (2011). Tet Proteins Can Convert 5-Methylcytosine to 5-Formylcytosine and 5-Carboxylcytosine. Science.

[11] Li Y, Nagai H, Ohno T, Yuge M, Hatano S, Ito E, Mori N, Saito H, Kinoshita T (2002). Aberrant DNA methylation of p57(KIP2) gene in the promoter region in lymphoid malignancies of B-cell phenotype. Blood.

[12] Wutz A, Smrzka OW, Schweifer N, Schellander K, Wagner EF, Barlow DP (1997). Imprinted expression of the Igf2r gene depends on an intronic CpG island. Nature.

[13] Robertson KD, Ait-Si-Ali S, Yokochi T, Wade PA, Jones PL, Wolffe AP (2000). DNMT1 forms a complex with Rb, E2F1 and HDAC1 and represses transcription from E2F-responsive promoters. Nature genetics.

[14] Nakamura M, Watanabe T, Yonekawa Y, Kleihues P, Ohgaki H (2001). Promoter methylation of the DNA repair gene MGMT in astrocytomas is frequently associated with G:C —> A:T mutations of the TP53 tumor suppressor gene. Carcinogenesis.

[15] Szulwach KE, Li X, Li Y, Song C-X, Wu H, Dai Q, Irier H, Upadhyay AK, Gearing M, Levey AI, Vasanthakumar A, Godley LA, Chang Q, Cheng X, He C, Jin P (2011). 5-hmC-mediated epigenetic dynamics during postnatal neurodevelopment and aging. Nature neuroscience.

[16] Xu Y, Wu F, Tan L, Kong L, Xiong L, Deng J, Barbera AJ, Zheng L, Zhang H, Huang S, Min J, Nicholson T, Chen T, Xu G, Shi Y, Zhang K (2011). Genome-wide regulation of 5hmC, 5mC, and gene expression by Tet1 hydroxylase in mouse embryonic stem cells. Molecular cell.

[17] Ito S, D'Alessio AC, Taranova OV, Hong K, Sowers LC, Zhang Y (2010). Role of Tet proteins in 5mC to 5hmC conversion, ES-cell self-renewal and inner cell mass specification. Nature.

[18] Ko M, Huang Y, Jankowska AM, Pape UJ, Tahiliani M, Bandukwala HS, An J, Lamperti ED, Koh KP, Ganetzky R, Liu XS, Aravind L, Agarwal S, Maciejewski JP, Rao A (2010). Impaired hydroxylation of 5-methylcytosine in myeloid cancers with mutant TET2. Nature.

[19] Mellen M, Ayata P, Dewell S, Kriaucionis S, Heintz N (2012). MeCP2 binds to 5hmC enriched within active genes and accessible chromatin in the nervous system. Cell.

[20] Anand P, Kunnumakkara AB, Sundaram C, Harikumar KB, Tharakan ST, Lai OS, Sung B, Aggarwal BB (2008). Cancer is a preventable disease that requires major lifestyle changes. Pharm Res.

[21] Hirano M, Satake W, Ihara K, Tsuge I, Kondo S, Saida K, Betsui H, Okubo K, Sakamoto H, Ueno S, Ikuno Y, Ishihara R, Iwahashi H, Ohishi M, Mano T, Yamashita T (2015). The First Nationwide Survey and Genetic Analyses of Bardet-Biedl Syndrome in Japan. PLoS ONE.

[22] Meindl A, Ditsch N, Kast K, Rhiem K, Schmutzler RK (2011). Hereditary breast and ovarian cancer: new genes, new treatments, new concepts. Dtsch Arztebl Int.

[23] Waddington CH (2012). The epigenotype. 1942. Int J Epidemiol.

[24] O'Leary K, Shia A, Cavicchioli F, Haley V, Comino A, Merlano M, Mauri F, Walter K, Lackner M, Wischnewsky MB, Crook T, Lo Nigro C, Schmid P (2015). Identification of Endoglin as an epigenetically regulated tumour-suppressor gene in lung cancer. Br J Cancer.

[25] Xue K, Gu JJ, Zhang Q, Mavis C, Hernandez-Ilizaliturri FJ, Czuczman MS, Guo Y (2016). Vorinostat, a histone deacetylase (HDAC) inhibitor, promotes cell cycle arrest and re-sensitizes rituximab- and chemo-resistant lymphoma cells to chemotherapy agents. J Cancer Res Clin Oncol.

[26] Sun C, Liu Z, Li S, Yang C, Xue R, Xi Y, Wang L, Wang S, He Q, Huang J, Xie S, Jiang W, Li D (2015). Down-regulation of c-Met and Bcl2 by microRNA-206, activates apoptosis, and inhibits tumor cell proliferation, migration and colony formation. Oncotarget.

[27] Ehrlich M (2002). DNA methylation in cancer: too much, but also too little. Oncogene.

[28] Kim JT, Li J, Song J, Lee EY, Weiss HL, Townsend CM, Evers BM (2015). Differential expression and tumorigenic function of neurotensin receptor 1 in neuroendocrine tumor cells. Oncotarget.

[29] Lando M, Fjeldbo CS, Wilting SM, B CS, Aarnes EK, Forsberg MF, Kristensen GB, Steenbergen RD, Lyng H (2015). Interplay between promoter methylation and chromosomal loss in gene silencing at 3p11-p14 in cervical cancer. Epigenetics.

[30] Abdelmaksoud-Dammak R, Saadallah-Kallel A, Miladi-Abdennadher I, Ayedi L, Khabir A, Sallemi-Boudawara T, Frikha M, Daoud J, Mokdad-Gargouri R (2016). CpG methylation of ubiquitin carboxyl-terminal hydrolase 1 (UCHL1) and P53 mutation pattern in sporadic colorectal cancer. Tumour Biol.

[31] Lin CH, Hsieh SY, Sheen IS, Lee WC, Chen TC, Shyu WC, Liaw YF (2001). Genome-wide hypomethylation in hepatocellular carcinogenesis. Cancer research.

[32] Ehrlich M, Jiang G, Fiala E, Dome JS, Yu MC, Long TI, Youn B, Sohn O-S, Widschwendter M, Tomlinson GE, Chintagumpala M, Champagne M, Parham D, Liang G, Malik K, Laird PW (2002). Hypomethylation and hypermethylation of DNA in Wilms tumors. Oncogene.

[33] Penn NW, Suwalski R, O'Riley C, Bojanowski K, Yura R (1972). The presence of 5-hydroxymethylcytosine in animal deoxyribonucleic acid. The Biochemical journal.

[34] Lian CG, Xu Y, Ceol C, Wu F, Larson A, Dresser K, Xu W, Tan L, Hu Y, Zhan Q, Lee CW, Hu D, Lian BQ, Kleffel S, Yang Y, Neiswender J (2012). Loss of 5-hydroxymethylcytosine is an epigenetic hallmark of melanoma. Cell.

[35] Kudo Y, Tateishi K, Yamamoto K, Yamamoto S, Asaoka Y, Ijichi H, Nagae G, Yoshida H, Aburatani H, Koike K (2012). Loss of 5-hydroxymethylcytosine is accompanied with malignant cellular transformation. Cancer Sci.

[36] Yang Q, Wu K, Ji M, Jin W, He N, Shi B, Hou P (2013). Decreased 5-hydroxymethylcytosine (5-hmC) is an independent poor prognostic factor in gastric cancer patients. Journal of biomedical nanotechnology.

[37] Yang H, Liu Y, Bai F, Zhang JY, Ma SH, Liu J, Xu ZD, Zhu HG, Ling ZQ, Ye D, Guan KL, Xiong Y (2013). Tumor development is associated with decrease of TET gene expression and 5-methylcytosine hydroxylation. Oncogene.

[38] Cimmino L, Dawlaty MM, Ndiaye-Lobry D, Yap YS, Bakogianni S, Yu Y, Bhattacharyya S, Shaknovich R, Geng H, Lobry C, Mullenders J, King B, Trimarchi T, Aranda-Orgilles B, Liu C, Shen S (2015). TET1 is a tumor suppressor of hematopoietic malignancy. Nature immunology.

[39] Tsai T, Davalath S, Rankin C, Radich JP, Head D, Appelbaum FR, Boldt DH (1996). Tumor suppressor gene alteration in adult acute lymphoblastic leukemia (ALL). Analysis of retinoblastoma (Rb) and p53 gene expression in lymphoblasts of patients with de novo, relapsed, or refractory ALL treated in Southwest Oncology Group studies. Leukemia.

[40] Tawana Kiran, Fitzgibbon J (1993). CEBPA-Associated Familial Acute Myeloid Leukemia (AML) BTI - GeneReviews(R). GeneReviews.

[41] Cheng J, Haas M (1990). Frequent mutations in the p53 tumor suppressor gene in human leukemia T-cell lines. Mol Cell Biol.

[42] Daley GQ, Van Etten RA, Baltimore D (1990). Induction of chronic myelogenous leukemia in mice by the P210bcr/abl gene of the Philadelphia chromosome. Science.

[43] Gauwerky CE, Croce CM (1993). Chromosomal translocations in leukaemia. Semin Cancer Biol.

[44] Viguie F, Aboura A, Bouscary D, Ramond S, Delmer A, Tachdjian G, Marie JP, Casadevall N (2005). Common 4q24 deletion in four cases of hematopoietic malignancy: early stem cell involvement?. Leukemia.

[45] Figueroa ME, Abdel-Wahab O, Lu C, Ward PS, Patel J, Shih A, Li Y, Bhagwat N, Vasanthakumar A, Fernandez HF, Tallman MS, Sun Z, Wolniak K, Peeters JK, Liu W, Choe SE (2010). Leukemic IDH1 and IDH2 mutations result in a hypermethylation phenotype, disrupt TET2 function, and impair hematopoietic differentiation. Cancer Cell.

[46] Kroeze LI, Aslanyan MG, van Rooij A, Koorenhof-Scheele TN, Massop M, Carell T, Boezeman JB, Marie JP, Halkes CJ, de Witte T, Huls G, Suciu S, Wevers RA, van der Reijden BA, Jansen JH (2014). Characterization of acute myeloid leukemia based on levels of global hydroxymethylation. Blood.

[47] Ogawara Y, Katsumoto T, Aikawa Y, Shima Y, Kagiyama Y, Soga T, Matsunaga H, Seki T, Araki K, Kitabayashi I (2015). IDH2 and NPM1 Mutations Cooperate to Activate Hoxa9/Meis1 and Hypoxia Pathways in Acute Myeloid Leukemia. Cancer research.

[48] Chotirat S, Thongnoppakhun W, Wanachiwanawin W, Auewarakul CU (2015). Acquired somatic mutations of isocitrate dehydrogenases 1 and 2 (IDH1 and IDH2) in preleukemic disorders. Blood cells, molecules & diseases.

[49] Sardina JL, Graf T (2015). A new path to leukemia with WIT. Molecular cell.

[50] Rampal R, Alkalin A, Madzo J, Vasanthakumar A, Pronier E, Patel J, Li Y, Ahn J, Abdel-Wahab O, Shih A, Lu C, Ward PS, Tsai JJ, Hricik T, Tosello V, Tallman JE (2014). DNA hydroxymethylation profiling reveals that WT1 mutations result in loss of TET2 function in acute myeloid leukemia. Cell reports.

[51] Wang Y, Xiao M, Chen X, Chen L, Xu Y, Lv L, Wang P, Yang H, Ma S, Lin H, Jiao B, Ren R, Ye D, Guan K-L, Xiong Y (2015). WT1 recruits TET2 to regulate its target gene expression and suppress leukemia cell proliferation. Molecular cell.

[52] McCarty G, Loeb DM (2015). Hypoxia-sensitive epigenetic regulation of an antisense-oriented lncRNA controls WT1 expression in myeloid leukemia cells. PLoS ONE.

[53] Nakagawa T, Lv L, Nakagawa M, Yu Y, Yu C, D'Alessio AC, Nakayama K, Fan H-Y, Chen X, Xiong Y (2015). CRL4(VprBP) E3 ligase promotes monoubiquitylation and chromatin binding of TET dioxygenases. Molecular cell.

[54] Rasmussen KD, Helin K (2015). TET1: an epigenetic guardian of lymphomagenesis. Nature immunology.

[55] Cimmino L, Dawlaty MM, Ndiaye-Lobry D, Yap YS, Bakogianni S, Yu Y, Bhattacharyya S, Shaknovich R, Geng H, Lobry C, Mullenders J, King B, Trimarchi T, Aranda-Orgilles B, Liu C, Shen S (2015). Erratum: TET1 is a tumor suppressor of hematopoietic malignancy. Nature immunology.

[56] Quivoron C, Couronne L, Della Valle V, Lopez CK, Plo I, Wagner-Ballon O, Do Cruzeiro M, Delhommeau F, Arnulf B, Stern MH, Godley L, Opolon P, Tilly H, Solary E, Duffourd Y, Dessen P (2011). TET2 inactivation results in pleiotropic hematopoietic abnormalities in mouse and is a recurrent event during human lymphomagenesis. Cancer Cell.

[57] Asmar F, Punj V, Christensen J, Pedersen MT, Pedersen A, Nielsen AB, Hother C, Ralfkiaer U, Brown P, Ralfkiaer E, Helin K, Gronbak K (2013). Genome-wide profiling identifies a DNA methylation signature that associates with TET2 mutations in diffuse large B-cell lymphoma. Haematologica.

[58] Cimmino L, Dawlaty MM, Ndiaye-Lobry D, Yap YS, Bakogianni S, Yu Y, Bhattacharyya S, Shaknovich R, Geng H, Lobry C, Mullenders J, King B, Trimarchi T, Aranda-Orgilles B, Liu C, Shen S (2015). TET1 is a tumor suppressor of hematopoietic malignancy. Nat Immunol.

[59] Lemonnier F, Couronne L, Parrens M, Jais J-P, Travert M, Lamant L, Tournillac O, Rousset T, Fabiani B, Cairns RA, Mak T, Bastard C, Bernard OA, de Leval L, Gaulard P (2012). Recurrent TET2 mutations in peripheral T-cell lymphomas correlate with TFH-like features and adverse clinical parameters. Blood.

[60] Quivoron C, Couronne L, Della Valle V, Lopez CK, Plo I, Wagner-Ballon O, Do Cruzeiro M, Delhommeau F, Arnulf B, Stern M-H, Godley L, Opolon P, Tilly H, Solary E, Duffourd Y, Dessen P (2011). TET2 inactivation results in pleiotropic hematopoietic abnormalities in mouse and is a recurrent event during human lymphomagenesis. Cancer cell.

[61] Couronne L, Bastard C, Bernard OA (2012). TET2 and DNMT3A mutations in human T-cell lymphoma. The New England journal of medicine.

[62] Odejide O, Weigert O, Lane AA, Toscano D, Lunning MA, Kopp N, Kim S, van Bodegom D, Bolla S, Schatz JH, Teruya-Feldstein J, Hochberg E, Louissaint A, Dorfman D, Stevenson K, Rodig SJ (2014). A targeted mutational landscape of angioimmunoblastic T-cell lymphoma. Blood.

[63] Sakata-Yanagimoto M, Enami T, Yoshida K, Shiraishi Y, Ishii R, Miyake Y, Muto H, Tsuyama N, Sato-Otsubo A, Okuno Y, Sakata S, Kamada Y, Nakamoto-Matsubara R, Tran NB, Izutsu K, Sato Y (2014). Somatic RHOA mutation in angioimmunoblastic T cell lymphoma. Nature genetics.

[64] Galm O, Wilop S, Reichelt J, Jost E, Gehbauer G, Herman JG, Osieka R (2004). DNA methylation changes in multiple myeloma. Leukemia.

[65] Galm O, Yoshikawa H, Esteller M, Osieka R, Herman JG (2003). SOCS-1, a negative regulator of cytokine signaling, is frequently silenced by methylation in multiple myeloma. Blood.

[66] Troppan K, Hofer S, Wenzl K, Lassnig M, Pursche B, Steinbauer E, Wiltgen M, Zulus B, Renner W, Beham-Schmid C, Deutsch A, Neumeister P (2015). Frequent down regulation of the tumor suppressor gene a20 in multiple myeloma. PLoS ONE.

[67] Zhang Q, Wang LQ, Wong KY, Li ZY, Chim CS (2015). Infrequent DNA methylation of miR-9-1 and miR-9-3 in multiple myeloma. Journal of clinical pathology.

[68] Heuck CJ, Mehta J, Bhagat T, Gundabolu K, Yu Y, Khan S, Chrysofakis G, Schinke C, Tariman J, Vickrey E, Pulliam N, Nischal S, Zhou L, Bhattacharyya S, Meagher R, Hu C (2013). Myeloma is characterized by stage-specific alterations in DNA methylation that occur early during myelomagenesis. Journal of immunology.

[69] Agirre X, Castellano G, Pascual M, Heath S, Kulis M, Segura V, Bergmann A, Esteve A, Merkel A, Raineri E, Agueda L, Blanc J, Richardson D, Clarke L, Datta A, Russinol N (2015). Whole-epigenome analysis in multiple myeloma reveals DNA hypermethylation of B cell-specific enhancers. Genome research.

[70] Miyamoto Y, Yoshida N, Yamamoto M, Oda S, Watanabe M, Nakao M, Baba H (2015). TET family proteins and 5-hydroxymethylcytosine in esophageal squamous cell carcinoma. Oncotarget.

[71] Kudo Y, Tateishi K, Yamamoto K, Yamamoto S, Asaoka Y, Ijichi H, Nagae G, Yoshida H, Aburatani H, Koike K (2012). Loss of 5-hydroxymethylcytosine is accompanied with malignant cellular transformation. Cancer science.

[72] Frycz BA, Murawa D, Borejsza-Wysocki M, Marciniak R, Murawa P, Drews M, Kolodziejczak A, Tomela K, Jagodzinski PP (2014). Decreased expression of ten-eleven translocation 1 protein is associated with some clinicopathological features in gastric cancer. Biomedicine & Pharmacotherapy.

[73] Du C, Kurabe N, Matsushima Y, Suzuki M, Kahyo T, Ohnishi I, Tanioka F, Tajima S, Goto M, Yamada H, Tao H, Shinmura K, Konno H, Sugimura H (2015). Robust quantitative assessments of cytosine modifications and changes in the expressions of related enzymes in gastric cancer. Gastric cancer.

[74] Park J-L, Kwon O-H, Song KS, Kim S-Y, Kim YS (2014). Abstract 394: TET1, which act as an tumor suppressor gene, is suppressed by DNA hypermethylation in gastric cancer. Cancer Research.

[75] Fu HL, Ma Y, Lu LG, Hou P, Li BJ, Jin WL, Cui DX (2014). TET1 exerts its tumor suppressor function by interacting with p53-EZH2 pathway in gastric cancer. J Biomed Nanotechnol.

[76] Li W, Liu M (2011). Distribution of 5-hydroxymethylcytosine in different human tissues. Journal of nucleic acids.

[77] Hur K, Cejas P, Feliu J, Moreno-Rubio J, Burgos E, Boland CR, Goel A (2014). Hypomethylation of long interspersed nuclear element-1 (LINE-1) leads to activation of proto-oncogenes in human colorectal cancer metastasis. Gut.

[78] Ichimura N, Shinjo K, An B, Shimizu Y, Yamao K, Ohka F, Katsushima K, Hatanaka A, Tojo M, Yamamoto E, Suzuki H, Ueda M, Kondo Y (2015). Aberrant TET1 Methylation Closely Associated with CpG Island Methylator Phenotype in Colorectal Cancer. Cancer prevention research (Philadelphia, Pa).

[79] Neri F, Dettori D, Incarnato D, Krepelova A, Rapelli S, Maldotti M, Parlato C, Paliogiannis P, Oliviero S (2015). TET1 is a tumour suppressor that inhibits colon cancer growth by derepressing inhibitors of the WNT pathway. Oncogene.

[80] Uribe-Lewis S, Stark R, Carroll T, Dunning MJ, Bachman M, Ito Y, Stojic L, Halim S, Vowler SL, Lynch AG, Delatte B, de Bony EJ, Colin L, Defrance M, Krueger F, Silva A-L (2015). 5-hydroxymethylcytosine marks promoters in colon that resist DNA hypermethylation in cancer. Genome biology.

[81] Zhao XQ, Zhang YF, Xia YF, Zhou ZM, Cao YQ (2015). Promoter demethylation of nuclear factor-erythroid 2-related factor 2 gene in drug-resistant colon cancer cells. Oncology Letters.

[82] Kang KA, Piao MJ, Kim KC, Kang HK, Chang WY, Park IC, Keum YS, Surh YJ, Hyun JW (2014). Epigenetic modification of Nrf2 in 5-fluorouracil-resistant colon cancer cells: involvement of TET-dependent DNA demethylation. Cell death & disease.

[83] Kraus TF, Globisch D, Wagner M, Eigenbrod S, Widmann D, Munzel M, Muller M, Pfaffeneder T, Hackner B, Feiden W, Schuller U, Carell T, Kretzschmar HA (2012). Low values of 5-hydroxymethylcytosine (5hmC), the “sixth base,” are associated with anaplasia in human brain tumors. Int J Cancer.

[84] Jin SG, Jiang Y, Qiu R, Rauch TA, Wang Y, Schackert G, Krex D, Lu Q, Pfeifer GP (2011). 5-Hydroxymethylcytosine is strongly depleted in human cancers but its levels do not correlate with IDH1 mutations. Cancer Res.

[85] Orr BA, Haffner MC, Nelson WG, Yegnasubramanian S, Eberhart CG (2012). Decreased 5-hydroxymethylcytosine is associated with neural progenitor phenotype in normal brain and shorter survival in malignant glioma. PLoS One.

[86] Turcan S, Rohle D, Goenka A, Walsh LA, Fang F, Yilmaz E, Campos C, Fabius AWM, Lu C, Ward PS, Thompson CB, Kaufman A, Guryanova O, Levine R, Heguy A, Viale A (2012). IDH1 mutation is sufficient to establish the glioma hypermethylator phenotype. Nature.

[87] Muller T, Gessi M, Waha A, Isselstein LJ, Luxen D, Freihoff D, Freihoff J, Becker A, Simon M, Hammes J, Denkhaus D, zur Muhlen A, Pietsch T, Waha A (2012). Nuclear Exclusion of TET1 Is Associated with Loss of 5-Hydroxymethylcytosine in IDH1 Wild-Type Gliomas. American Journal of Pathology.

[88] Orr BA, Haffner MC, Nelson WG, Yegnasubramanian S, Eberhart CG (2012). Decreased 5-hydroxymethylcytosine is associated with neural progenitor phenotype in normal brain and shorter survival in malignant glioma. PLoS ONE.

[89] Kraus Theo F. J., Kolck Gesa, Greiner Andrea, Schierl Katharina, Guibourt Virginie, Kretzschmar Hans A. (2015). Loss of 5-hydroxymethylcytosine and intratumoral heterogeneity as an epigenomic hallmark of glioblastoma. Tumor Biol.

[90] Kraus TFJ, Greiner A, Steinmaurer M, Dietinger V, Guibourt V, Kretzschmar HA (2015). Genetic Characterization of Ten-Eleven-Translocation Methylcytosine Dioxygenase Alterations in Human Glioma. Journal of Cancer.

[91] Gambichler T, Sand M, Skrygan M (2013). Loss of 5-hydroxymethylcytosine and ten-eleven translocation 2 protein expression in malignant melanoma. Melanoma research.

[92] Hon GC, Hawkins RD, Caballero OL, Lo C, Lister R, Pelizzola M, Valsesia A, Ye Z, Kuan S, Edsall LE, Camargo AA, Stevenson BJ, Ecker JR, Bafna V, Strausberg RL, Simpson AJ (2012). Global DNA hypomethylation coupled to repressive chromatin domain formation and gene silencing in breast cancer. Genome research.

[93] Jawert F, Hasseus B, Kjeller G, Magnusson B, Sand L, Larsson L (2013). Loss of 5-hydroxymethylcytosine and TET2 in oral squamous cell carcinoma. Anticancer research.

[94] Xie K, Liu J, Chen J, Dong J, Ma H, Liu Y, Hu Z (2014). Methylation-associated silencing of microRNA-34b in hepatocellular carcinoma cancer. Gene.

[95] Santos F, Hendrich B, Reik W, Dean W (2002). Dynamic reprogramming of DNA methylation in the early mouse embryo. Developmental Biology.

[96] Frank D, Keshet I, Shani M, Levine A, Razin A, Cedar H (1991). Demethylation of Cpg Islands in Embryonic-Cells. Nature.

[97] Mayer W, Niveleau A, Walter J, Fundele R, Haaf T (2000). Embryogenesis - Demethylation of the zygotic paternal genome. Nature.

[98] Smith ZD, Chan MM, Mikkelsen TS, Gu HC, Gnirke A, Regev A, Meissner A (2012). A unique regulatory phase of DNA methylation in the early mammalian embryo. Nature.

[99] Gaudet F, Rideout WM, Meissner A, Dausman J, Leonhardt H, Jaenisch R (2004). Dnmt1 expression in pre- and postimplantation embryogenesis and the maintenance of IAP silencing. Molecular and Cellular Biology.

[100] Biniszkiewicz D, Gribnau J, Ramsahoye B, Gaudet F, Eggan K, Humpherys D, Mastrangelo MA, Jun Z, Walter J, Jaenisch R (2002). Dnmt1 overexpression causes genomic hypermethylation, loss of imprinting, and embryonic lethality. Molecular and Cellular Biology.

[101] Howell CY, Bestor TH, Ding F, Latham KE, Mertineit C, Trasler JM, Chaillet JR (2001). Genomic imprinting disrupted by a maternal effect mutation in the Dnmt1 gene. Cell.

[102] Hackett JA, Sengupta R, Zylicz JJ, Murakami K, Lee C, Down TA, Surani MA (2013). Germline DNA Demethylation Dynamics and Imprint Erasure Through 5-Hydroxymethylcytosine. Science.

[103] Kang J, Lienharda M, Pastor WA, Chawla A, Novotny M, Tsagaratou A, Lasken RS, Thompson EC, Surani MA, Koralov SB, Kalantry S, Chavez L, Rao A (2015). Simultaneous deletion of the methylcytosine oxidases Tet1 and Tet3 increases transcriptome variability in early embryogenesis. Proceedings of the National Academy of Sciences of the United States of America.

[104] Cao ZB, Zhou NR, Zhang Y, Zhang YL, Wu RH, Li YS, Zhang YH, Li N (2014). Dynamic reprogramming of 5-hydroxymethylcytosine during early porcine embryogenesis. Theriogenology.

[105] Fritz EL, Papavasiliou FN (2010). Cytidine deaminases: AIDing DNA demethylation?. Genes Dev.

[106] He XJ, Chen T, Zhu JK (2011). Regulation and function of DNA methylation in plants and animals. Cell Res.

[107] Inoue A, Zhang Y (2011). Replication-dependent loss of 5-hydroxymethylcytosine in mouse preimplantation embryos. Science.

[108] Kohli RM, Zhang Y (2013). TET enzymes, TDG and the dynamics of DNA demethylation. Nature.

[109] Inoue A, Shen L, Dai Q, He C, Zhang Y (2011). Generation and replication-dependent dilution of 5fC and 5caC during mouse preimplantation development. Cell Res.

[110] Dawlaty MM, Ganz K, Powell BE, Hu YC, Markoulaki S, Cheng AW, Gao Q, Kim J, Choi SW, Page DC, Jaenisch R (2011). Tet1 is dispensable for maintaining pluripotency and its loss is compatible with embryonic and postnatal development. Cell Stem Cell.

[111] Etchegaray JP, Chavez L, Huang Y, Ross KN, Choi J, Martinez-Pastor B, Walsh RM, Sommer CA, Lienhard M, Gladden A, Kugel S, Silberman DM, Ramaswamy S, Mostoslavsky G, Hochedlinger K, Goren A (2015). The histone deacetylase SIRT6 controls embryonic stem cell fate via TET-mediated production of 5-hydroxymethylcytosine. Nat Cell Biol.

[112] Kang J, Lienhard M, Pastor WA, Chawla A, Novotny M, Tsagaratou A, Lasken RS, Thompson EC, Surani MA, Koralov SB, Kalantry S, Chavez L, Rao A (2015). Simultaneous deletion of the methylcytosine oxidases Tet1 and Tet3 increases transcriptome variability in early embryogenesis. Proceedings of the National Academy of Sciences of the United States of America.

[113] Dawlaty MM, Breiling A, Le T, Barrasa MI, Raddatz G, Gao Q, Powell BE, Cheng AW, Faull KF, Lyko F, Jaenisch R (2014). Loss of Tet enzymes compromises proper differentiation of embryonic stem cells. Developmental cell.

[114] Liu L, Mao SQ, Ray C, Zhang Y, Bell FT, Ng SF, Xu GL, Li X (2015). Differential regulation of genomic imprinting by TET proteins in embryonic stem cells. Stem Cell Res.

[115] Okashita N, Kumaki Y, Ebi K, Nishi M, Okamoto Y, Nakayama M, Hashimoto S, Nakamura T, Sugasawa K, Kojima N, Takada T, Okano M, Seki Y (2014). PRDM14 promotes active DNA demethylation through the Ten-eleven translocation (TET)-mediated base excision repair pathway in embryonic stem cells. Development.

[116] Chiquoine AD (1954). The Identification, Origin, and Migration of the Primordial Germ Cells in the Mouse Embryo. Anatomical Record.

[117] Ginsburg M, Snow MH, McLaren A (1990). Primordial germ cells in the mouse embryo during gastrulation. Development.

[118] Zhao GQ, Garbers DL (2002). Male germ cell specification and differentiation. Developmental Cell.

[119] Shamblott MJ, Axelman J, Wang S, Bugg EM, Littlefield JW, Donovan PJ, Blumenthal PD, Huggins GR, Gearhart JD (1998). Derivation of pluripotent stem cells from cultured human primordial germ cells. Proceedings of the National Academy of Sciences of the United States of America.

[120] Lee J, Inoue K, Ono R, Ogonuki N, Kohda T, Kaneko-Ishino T, Ogura A, Ishino F (2002). Erasing genomic imprinting memory in mouse clone embryos produced from day 11. 5 primordial germ cells. Development.

[121] Yamazaki Y, Mann MRW, Lee SS, Marh J, McCarrey JR, Yanagimachi R, Bartolomei MS (2003). Reprogramming of primordial germ cells begins before migration into the genital ridge, making these cells inadequate donors for reproductive cloning. Proceedings of the National Academy of Sciences of the United States of America.

[122] Sasaki H, Matsui Y (2008). Epigenetic events in mammalian germ-cell development: reprogramming and beyond. Nature reviews Genetics.

[123] Saitou M, Kagiwada S, Kurimoto K (2012). Epigenetic reprogramming in mouse pre-implantation development and primordial germ cells. Development.

[124] Smallwood SA, Kelsey G (2012). De novo DNA methylation: a germ cell perspective. Trends Genet.

[125] Yamaguchi S, Hong K, Liu R, Shen L, Inoue A, Diep D, Zhang K, Zhang Y (2013). 5mC and 5hmC dynamics during PGC reprogramming and role of Tet1 in female meiosis. Epigenetics & Chromatin.

[126] Vincent JJ, Huang Y, Chen P-Y, Feng S, Calvopina JH, Nee K, Lee SA, Le T, Yoon AJ, Faull K, Fan G, Rao A, Jacobsen SE, Pellegrini M, Clark AT (2013). Stage-specific roles for tet1 and tet2 in DNA demethylation in primordial germ cells. Cell stem cell.

[127] Hajkova P, Jeffries SJ, Lee C, Miller N, Jackson SP, Surani MA (2010). Genome-wide reprogramming in the mouse germ line entails the base excision repair pathway. Science.

[128] Popp C, Dean W, Feng SH, Cokus SJ, Andrews S, Pellegrini M, Jacobsen SE, Reik W (2010). Genome-wide erasure of DNA methylation in mouse primordial germ cells is affected by AID deficiency. Nature.

[129] Calvopina JH, Cook H, Vincent JJ, Nee K, Clark AT (2015). The Aorta-Gonad-Mesonephros Organ Culture Recapitulates 5hmC Reorganization and Replication-Dependent and Independent Loss of DNA Methylation in the Germline. Stem Cells Dev.

[130] Feng J, Fan G (2009). The role of DNA methylation in the central nervous system and neuropsychiatric disorders. International review of neurobiology.

[131] Teter B, Rozovsky I, Krohn K, Anderson C, Osterburg H, Finch C (1996). Methylation of the glial fibrillary acidic protein gene shows novel biphasic changes during brain development. Glia.

[132] Devlin AM, Brain U, Austin J, Oberlander TF (2010). Prenatal exposure to maternal depressed mood and the MTHFR C677T variant affect SLC6A4 methylation in infants at birth. PLoS ONE.

[133] Guo JJU, Ma DKK, Mo H, Ball MP, Jang MH, Bonaguidi MA, Balazer JA, Eaves HL, Xie B, Ford E, Zhang K, Ming GL, Gao Y, Song HJ (2011). Neuronal activity modifies the DNA methylation landscape in the adult brain. Nature Neuroscience.

[134] Costello JF (2003). DNA methylation in brain development and gliomagenesis. Frontiers in Bioscience.

[135] Kawai J, Hirotsune S, Hirose K, Fushiki S, Watanabe S, Hayashizaki Y (1993). Methylation Profiles of Genomic DNA of Mouse Developmental Brain Detected by Restriction Landmark Genomic Scanning (Rlgs) Method. Nucleic Acids Research.

[136] Fuchikami M, Morinobu S, Segawa M, Okamoto Y, Yamawaki S, Ozaki N, Inoue T, Kusumi I, Koyama T, Tsuchiyama K, Terao T (2011). DNA methylation profiles of the brain-derived neurotrophic factor (BDNF) gene as a potent diagnostic biomarker in major depression. PLoS One.

[137] Hutnick LK, Golshani P, Namihira M, Xue ZG, Matynia A, Yang XW, Silva AJ, Schweizer FE, Fan GP (2009). DNA hypomethylation restricted to the murine forebrain induces cortical degeneration and impairs postnatal neuronal maturation. Human Molecular Genetics.

[138] Wu H, Coskun V, Tao JF, Xie W, Ge WH, Yoshikawa K, Li E, Zhang Y, Sun YE (2010). Dnmt3a-Dependent Nonpromoter DNA Methylation Facilitates Transcription of Neurogenic Genes. Science.

[139] Kriaucionis S, Heintz N (2009). The Nuclear DNA Base 5-Hydroxymethylcytosine Is Present in Purkinje Neurons and the Brain. Science.

[140] Kriaucionis S, Heintz N (2009). The nuclear DNA base 5-hydroxymethylcytosine is present in Purkinje neurons and the brain. Science.

[141] Gross JA, Pacis A, Chen GG, Barreiro LB, Ernst C, Turecki G (2015). Characterizing 5-hydroxymethylcytosine in human prefrontal cortex at single base resolution. BMC genomics.

[142] Guo JU, Szulwach KE, Su Y, Li Y, Yao B, Xu Z, Shin JH, Xie B, Gao Y, Ming GL, Jin P, Song H (2014). Genome-wide antagonism between 5-hydroxymethylcytosine and DNA methylation in the adult mouse brain. Front Biol (Beijing).

[143] Jin SG, Wu XW, Li AX, Pfeifer GP (2011). Genomic mapping of 5-hydroxymethylcytosine in the human brain. Nucleic Acids Research.

[144] Zheng T, Lv Q, Lei X, Yin X, Zhang B (2015). Spatial distribution of 5-hydroxymethyl cytosine in rat brain and temporal distribution in striatum. Neurochemical research.

[145] Wang T, Pan Q, Lin L, Szulwach KE, Song C-X, He C, Wu H, Warren ST, Jin P, Duan R, Li X (2012). Genome-wide DNA hydroxymethylation changes are associated with neurodevelopmental genes in the developing human cerebellum. Human molecular genetics.

[146] Zhang RR, Cui QY, Murai K, Lim YC, Smith ZD, Jin SN, Ye P, Rosa L, Lee YK, Wu HP, Liu W, Xu ZM, Yang L, Ding YQ, Tang FC, Meissner A (2013). Tet1 Regulates Adult Hippocampal Neurogenesis and Cognition. Cell Stem Cell.

[147] Colquitt BM, Allen WE, Barnea G, Lomvardas S (2013). Alteration of genic 5-hydroxymethylcytosine patterning in olfactory neurons correlates with changes in gene expression and cell identity. Proceedings of the National Academy of Sciences of the United States of America.

[148] Bose R, Spulber S, Kilian P, Heldring N, Lonnerberg P, Johnsson A, Conti M, Hermanson O, Ceccatelli S (2015). Tet3 mediates stable glucocorticoid-induced alterations in DNA methylation and Dnmt3a/Dkk1 expression in neural progenitors. Cell Death Dis.

[149] Dzitoyeva S, Chen H, Manev H (2012). Effect of aging on 5-hydroxymethylcytosine in brain mitochondria. Neurobiology of Aging.

[150] He X-B, Kim M, Kim S-Y, Yi S-H, Rhee Y-H, Kim T, Lee E-H, Park C-H, Dixit S, Harrison FE, Lee S-H (2015). Vitamin C facilitates dopamine neuron differentiation in fetal midbrain through TET1- and JMJD3-dependent epigenetic control manner. Stem cells.

[151] Wang F, Yang Y, Lin X, Wang J-Q, Wu Y-S, Xie W, Wang D, Zhu S, Liao Y-Q, Sun Q, Yang Y-G, Luo H-R, Guo C, Han C, Tang T-S (2013). Genome-wide loss of 5-hmC is a novel epigenetic feature of Huntington's disease. Human molecular genetics.

[152] Coppieters N, Dieriks BV, Lill C, Faull RLM, Curtis MA, Dragunow M (2014). Global changes in DNA methylation and hydroxymethylation in Alzheimer's disease human brain. Neurobiology of aging.

[153] Zhubi A, Chen Y, Dong E, Cook EH, Guidotti A, Grayson DR (2014). Increased binding of MeCP2 to the GAD1 and RELN promoters may be mediated by an enrichment of 5-hmC in autism spectrum disorder (ASD) cerebellum. Translational psychiatry.

[154] Dong E, Dzitoyeva SG, Matrisciano F, Tueting P, Grayson DR, Guidotti A (2015). Brain-Derived Neurotrophic Factor Epigenetic Modifications Associated with Schizophrenia-like Phenotype Induced by Prenatal Stress in Mice. Biological psychiatry.

[155] Greco C, Kunderfranco P, Carullo P, Papait R, Condorelli G (2014). P356Dynamic nature of the methylation landscape of the heart. Cardiovascular Research.

[156] Terragni J, Zhang G, Sun Z, Pradhan S, Song L, Crawford GE, Lacey M, Ehrlich M (2014). Notch signaling genes: myogenic DNA hypomethylation and 5-hydroxymethylcytosine. Epigenetics.

[157] Wendler C, Poulsen R, Fang X (2014). Caffeine induces both short-term and long-term effects on gene expression and DNA methylation in the mouse heart (542.3). The FASEB Journal.

[158] Fang X, Robinson J, Wang-Hu J, Jiang L, Freeman DA, Rivkees SA, Wendler CC (2015). cAMP induces hypertrophy and alters DNA methylation in HL-1 cardiomyocytes. American Journal of Physiology - Cell Physiology.

[159] Liu R, Jin Y, Tang WH, Qin L, Zhang X, Tellides G, Hwa J, Yu J, Martin KA (2013). Ten-eleven translocation-2 (TET2) is a master regulator of smooth muscle cell plasticity. Circulation.

[160] Chiou HY, Huang WI, Su CL, Chang SF, Hsu YH, Chen CJ (1997). Dose-response relationship between prevalence of cerebrovascular disease and ingested inorganic arsenic. Stroke.

[161] Ma W, Zhao L, Yin K, Feng D, Yang F, Liang J, Chen H, Bi C, Li X, Wang Y, Cai B (2015). Effects of arsenic trioxide on proliferation, paracrine and migration of cardiac progenitor cells. International journal of cardiology.

[162] Chu W, Li C, Qu X, Zhao D, Wang X, Yu X, Cai F, Liang H, Zhang Y, Zhao X, Li B, Qiao G, Dong D, Lu Y, Du Z, Yang B (2012). Arsenic-induced interstitial myocardial fibrosis reveals a new insight into drug-induced long QT syndrome. Cardiovascular research.

[163] Zhang J, Mu X, Xu W, Martin FL, Alamdar A, Liu L, Tian M, Huang Q, Shen H (2014). Exposure to arsenic via drinking water induces 5-hydroxymethylcytosine alteration in rat. The Science of the total environment.

[164] Liu S, Jiang J, Li L, Amato NJ, Wang Z, Wang Y (2015). Arsenite Targets the Zinc Finger Domains of Tet Proteins and Inhibits Tet-Mediated Oxidation of 5-Methylcytosine. Environ Sci Technol.

[165] Tang M, Xu W, Wang Q, Xiao W, Xu R (2009). Potential of DNMT and its Epigenetic Regulation for Lung Cancer Therapy. Current Genomics.

[166] Liu CC, Fang TJ, Ou TT, Wu CC, Li RN, Lin YC, Lin CH, Tsai WC, Liu HW, Yen JH (2011). Global DNA methylation, DNMT1, and MBD2 in patients with rheumatoid arthritis. Immunol Lett.

[167] Wu CT, Wu CF, Lu CH, Lin CC, Chen WC, Lin PY, Chen MF (2011). Expression and function role of DNA methyltransferase 1 in human bladder cancer. Cancer.

[168] Okamura D, Matsuda A, Ishikawa M, Maeda T, Tanae K, Kohri M, Takahashi N, Kawai N, Asou N, Bessho M (2014). Hematologic improvements in a myelodysplastic syndromes with myelofibrosis (MDS-F) patient treated with azacitidine. Leuk Res Rep.

[169] Steensma DP (2009). Decitabine treatment of patients with higher-risk myelodysplastic syndromes. Leuk Res.

[170] Claxton D, Erba HP, Faderl S, Arellano M, Lyons RM, Kovacsovics T, Gabrilove J, Huebner D, Gandhi PJ, Kantarjian H (2012). Outpatient consolidation treatment with clofarabine in a phase 2 study of older adult patients with previously untreated acute myelogenous leukemia. Leuk Lymphoma.

[171] Kaminskas E, Farrell AT, Wang YC, Sridhara R, Pazdur R (2005). FDA drug approval summary: azacitidine (5-azacytidine, Vidaza) for injectable suspension. Oncologist.

[172] Huls G (2015). Azacitidine in AML: a treatment option?. Blood.

[173] Hahn NM, Bonney PL, Dhawan D, Jones DR, Balch C, Guo Z, Hartman-Frey C, Fang F, Parker HG, Kwon EM, Ostrander EA, Nephew KP, Knapp DW (2012). Subcutaneous 5-azacitidine treatment of naturally occurring canine urothelial carcinoma: a novel epigenetic approach to human urothelial carcinoma drug development. J Urol.

[174] Schneider-Stock R, Diab-Assef M, Rohrbeck A, Foltzer-Jourdainne C, Boltze C, Hartig R, Schönfeld P, Roessner A, Gali-Muhtasib H (2005). 5-Aza-cytidine is a potent inhibitor of DNA methyltransferase 3a and induces apoptosis in HCT-116 colon cancer cells via Gadd45- and p53-dependent mechanisms. J Pharmacol Exp Ther.

[175] Miyoshi E, Fujii J, Hayashi N, Ueda K, Towata T, Fusamoto H, Kamada T, Taniguchi N (1992). Enhancement of hepatitis-B surface-antigen expression by 5-azacytidine in a hepatitis-B-virus-transfected cell line. Int J Cancer.

[176] Christman JK (2002). 5-Azacytidine and 5-aza-2′-deoxycytidine as inhibitors of DNA methylation: mechanistic studies and their implications for cancer therapy. Oncogene.

[177] Malik P, Cashen AF (2014). Decitabine in the treatment of acute myeloid leukemia in elderly patients. Cancer Manag Res.

[178] Kantarjian H, Issa JP, Rosenfeld CS, Bennett JM, Albitar M, DiPersio J, Klimek V, Slack J, de Castro C, Ravandi F, Helmer R, Shen L, Nimer SD, Leavitt R, Raza A, Saba H (2006). Decitabine improves patient outcomes in myelodysplastic syndromes: results of a phase III randomized study. Cancer.

[179] Momparler RL, Bouffard DY, Momparler LF, Dionne J, Belanger K, Ayoub J (1997). Pilot phase I-II study on 5-aza-2′-deoxycytidine (Decitabine) in patients with metastatic lung cancer. Anticancer Drugs.

[180] Ritchie EK, Feldman EJ, Christos PJ, Rohan SD, Lagassa CB, Ippoliti C, Scandura JM, Carlson K, Roboz GJ (2013). Decitabine in patients with newly diagnosed and relapsed acute myeloid leukemia. Leuk Lymphoma.

[181] Nagasawa T, Zhang Q, Raghunath PN, Wong HY, El-Salem M, Szallasi A, Marzec M, Gimotty P, Rook AH, Vonderheid EC, Odum N, Wasik MA (2006). Multi-gene epigenetic silencing of tumor suppressor genes in T-cell lymphoma cells; delayed expression of the p16 protein upon reversal of the silencing. Leuk Res.

[182] Lemaire M, Momparler LF, Raynal NJ, Bernstein ML, Momparler RL (2009). Inhibition of cytidine deaminase by zebularine enhances the antineoplastic action of 5-aza-2′-deoxycytidine. Cancer Chemother Pharmacol.

[183] Zhou L, Cheng X, Connolly BA, Dickman MJ, Hurd PJ, Hornby DP (2002). Zebularine: a novel DNA methylation inhibitor that forms a covalent complex with DNA methyltransferases. J Mol Biol.

[184] Cheng JC, Yoo CB, Weisenberger DJ, Chuang J, Wozniak C, Liang G, Marquez VE, Greer S, Orntoft TF, Thykjaer T, Jones PA (2004). Preferential response of cancer cells to zebularine. Cancer Cell.

[185] Xue ZT, Sjögren HO, Salford LG, Widegren B (2012). An epigenetic mechanism for high, synergistic expression of indoleamine 2,3-dioxygenase 1 (IDO1) by combined treatment with zebularine and IFN-gamma: potential therapeutic use in autoimmune diseases. Molecular Immunology.

[186] Naeem N, Gul A, Ali A, Khan I, Salim A (2014). Abstract 15368: Zebularine Enhances Cardiomyogenic Differentiation Potential of both Mesenchymal Stem Cells and Mature Fibroblasts. Circulation.

[187] Lech-Maranda E, Korycka A, Robak T (2009). Clofarabine as a novel nucleoside analogue approved to treat patients with haematological malignancies: mechanism of action and clinical activity. Mini Rev Med Chem.

[188] Bonate PL, Arthaud L, Cantrell WR, Stephenson K, Secrist JA, Weitman S (2006). Discovery and development of clofarabine: a nucleoside analogue for treating cancer. Nat Rev Drug Discov.

[189] Zhang Y, Shahriar M, Zhang J, Ahmed SU, Lim SH (2009). Clofarabine induces hypomethylation of DNA and expression of Cancer-Testis antigens. Leuk Res.

[190] Stumpel DJ, Schneider P, Pieters R, Stam RW (2015). The potential of clofarabine in MLL-rearranged infant acute lymphoblastic leukaemia. Eur J Cancer.

[191] Klisovic RB, Stock W, Cataland S, Klisovic MI, Liu S, Blum W, Green M, Odenike O, Godley L, Burgt JV, Van Laar E, Cullen M, Macleod AR, Besterman JM, Reid GK, Byrd JC (2008). A phase I biological study of MG98, an oligodeoxynucleotide antisense to DNA methyltransferase 1, in patients with high-risk myelodysplasia and acute myeloid leukemia. Clin Cancer Res.

[192] Amato RJ, Stephenson J, Hotte S, Nemunaitis J, Belanger K, Reid G, Martell RE (2012). MG98, a second-generation DNMT1 inhibitor, in the treatment of advanced renal cell carcinoma. Cancer Invest.

[193] Brueckner B, Garcia Boy R, Siedlecki P, Musch T, Kliem HC, Zielenkiewicz P, Suhai S, Wiessler M, Lyko F (2005). Epigenetic reactivation of tumor suppressor genes by a novel small-molecule inhibitor of human DNA methyltransferases. Cancer Res.

[194] Savickiene J, Treigyte G, Jazdauskaite A, Borutinskaite VV, Navakauskiene R (2012). DNA methyltransferase inhibitor RG108 and histone deacetylase inhibitors cooperate to enhance NB4 cell differentiation and E-cadherin re-expression by chromatin remodelling. Cell Biol Int.

[195] Oh YS, Jeong SG, Cho GW (2015). Anti-senescence effects of DNA methyltransferase inhibitor RG108 in human bone marrow mesenchymal stromal cells. Biotechnol Appl Biochem.

[196] Blaschke K, Ebata KT, Karimi MM, Zepeda-Martinez JA, Goyal P, Mahapatra S, Tam A, Laird DJ, Hirst M, Rao A, Lorincz MC, Ramalho-Santos M (2013). Vitamin C induces Tet-dependent DNA demethylation and a blastocyst-like state in ES cells. Nature.

[197] Lin JR, Qin HH, Wu WY, He SJ, Xu JH (2014). Vitamin C protects against UV irradiation-induced apoptosis through reactivating silenced tumor suppressor genes p21 and p16 in a Tet-dependent DNA demethylation manner in human skin cancer cells. Cancer Biother Radiopharm.

